# Molecular cloning and biochemical characterization of a novel erythrose reductase from *Candida magnoliae *JH110

**DOI:** 10.1186/1475-2859-9-43

**Published:** 2010-06-08

**Authors:** Dae-Hee Lee, Ye-Ji Lee, Yeon-Woo Ryu, Jin-Ho Seo

**Affiliations:** 1 Department of Agricultural Biotechnology, Seoul National University, Seoul 151-921, Korea; 2 Department of Molecular Science and Technology, Ajou University, Suwon 442-749, Korea; 3 Department of Bioengineering, University of California San Diego, La Jolla, CA 92093, USA

## Abstract

**Background:**

Erythrose reductase (ER) catalyzes the final step of erythritol production, which is reducing erythrose to erythritol using NAD(P)H as a cofactor. ER has gained interest because of its importance in the production of erythritol, which has extremely low digestibility and approved safety for diabetics. Although ERs were purified and characterized from microbial sources, the entire primary structure and the corresponding DNA for ER still remain unknown in most of erythritol-producing yeasts. *Candida magnoliae *JH110 isolated from honeycombs produces a significant amount of erythritol, suggesting the presence of erythrose metabolizing enzymes. Here we provide the genetic sequence and functional characteristics of a novel NADPH-dependent ER from *C. magnoliae *JH110.

**Results:**

The gene encoding a novel ER was isolated from an osmophilic yeast *C. magnoliae *JH110. The ER gene composed of 849 nucleotides encodes a polypeptide with a calculated molecular mass of 31.4 kDa. The deduced amino acid sequence of ER showed a high degree of similarity to other members of the aldo-keto reductase superfamily including three ER isozymes from *Trichosporonoides megachiliensis *SNG-42. The intact coding region of ER from *C. magnoliae *JH110 was cloned, functionally expressed in *Escherichia coli *using a combined approach of gene fusion and molecular chaperone co-expression, and subsequently purified to homogeneity. The enzyme displayed a temperature and pH optimum at 42°C and 5.5, respectively. Among various aldoses, the *C. magnoliae *JH110 ER showed high specific activity for reduction of erythrose to the corresponding alcohol, erythritol. To explore the molecular basis of the catalysis of erythrose reduction with NADPH, homology structural modeling was performed. The result suggested that NADPH binding partners are completely conserved in the *C. magnoliae *JH110 ER. Furthermore, NADPH interacts with the side chains Lys252, Thr255, and Arg258, which could account for the enzyme's absolute requirement of NADPH over NADH.

**Conclusions:**

A novel ER enzyme and its corresponding gene were isolated from *C. magnoliae *JH110. The *C. magnoliae *JH110 ER with high activity and catalytic efficiency would be very useful for *in vitro *erythritol production and could be applied for the production of erythritol in other microorganisms, which do not produce erythritol.

## Background

Erythritol, a four-carbon polyol, occurs naturally in a wide variety of foods including many fruits and mushrooms as well as in fermented foods such as cheese, wine, beer, and soy sauce [1]. Because of its sweetness and mouthfeel-enhancing property, erythritol has been used as a low-calorie alternative sweetener in the food industry. It is also used for prevention of dental caries, since the bacteria that cause dental caries are unable to utilize erythritol as a carbon source [2]. In human, only about 10% of ingested erythritol is metabolized and excreted in the urine without changing insulin level [3,4]. Therefore, it has been used advantageously in special foods and pharmaceuticals for diabetic patients.

Erythritol can be synthesized from periodate-oxidized starch or dialdehyde starch by a high-temperature chemical reaction in the presence of a metal catalyst [5,6]. The chemical process has not been industrialized because it involves several intricate steps to get the erythritol and the starting material is expensive. Erythritol also can be synthesized by bioconversion that uses osmophilic yeasts and bacteria [7,8] and it has been produced commercially using an *Trichosporonoides megachiliensis *SNG-42 that produces erythritol in high yield [9]. This whole-cell biocatalytic production process is superior to chemical substrate conversion because it is a simpler process requiring only a few steps and is less expensive because a raw material is cheap and readily available. Recently, a high erythritol-producing yeast was isolated from honeycombs and identified as *Candida magnoliae *JH110 [10]. Various biological processes using *C. magnoliae *JH110 and its chemical mutant have been developed for maximizing erythritol production [11-13].

Erythrose reductase (ER) catalyzes the final step of erythritol production, which is reducing erythrose to erythritol with concomitant NAD(P)H oxidation [14,15]. It has been found in yeasts and bacteria with three isozymes in a yeast strain [16]. Although there have been several reports relating to the purification and characterization of ER from microbial sources [14,17,18], the entire primary structure and the corresponding DNA for ER still remain unknown in most of erythritol-producing yeasts. Such is the case with *C. magnoliae *JH110 [13], though one ER protein has been isolated from *C. magnoliae *KFCC-11023 [18], which is a mutant strain of *C. magnoliae *JH110 treated with ultraviolet irradiation and nitrosoguanidine for improved erythritol-producing ability [7]. Indeed, the ER-encoding genes were identified only recently in *T. megachiliensis *SNG-42 [16], which is one of the yeasts used for commercial production of erythritol [9].

In this study, we provide the genetic sequence and functional characteristics of a novel NADPH-dependent ER from *C. magnoliae *JH110, which has different enzymatic properties from the previously reported ER of *C. magnoliae *JH110 mutant strain [18]. Successful expression and characterization of the functional ER could offer a potential opportunity to produce erythritol in other microorganisms, which do not produce erythritol.

## Results

### *C. magnoliae *JH110 ER gene identification

**Figure 1:**
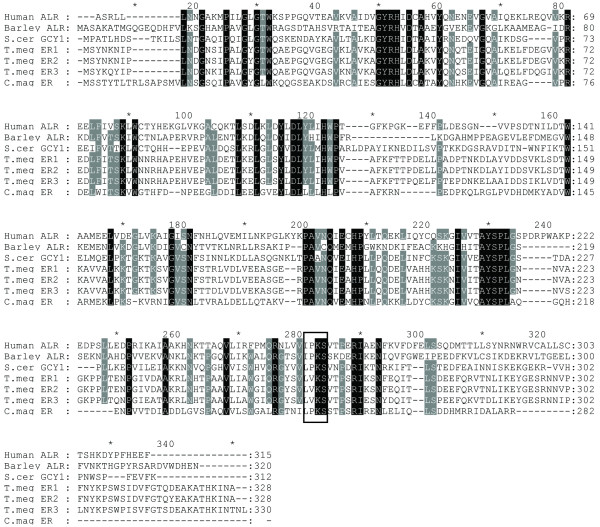
**Multiple alignment of the deduced amino acid sequence for the ER gene from *C. magnoliae *JH110 with other AKRs showing erythrose reduction activity**. AKRs are identified by their GenBank accession numbers: Human aldose reductase (ALR, AAB88851), barley ALR1 (CAA88322), *S. cerevisiae *GCY1 (CAA65512), *T. megachiliensis *SNG-42 ER1 (BAD90687), *T. megachiliensis *SNG-42 ER2 (BAD90688), *T. megachiliensis *SNG-42 ER3 (BAD90689), and *C. magnoliae *JH110 ER (FJ550210). The tetra-amino acid motif, IPKS, conserved among NADPH-dependent reductases is indicated by a box. Gray-shaded amino acids are conserved in at least six of the seven AKRs shown. Black-shaded amino acids are conserved in all sequences.

**Figure 2:**
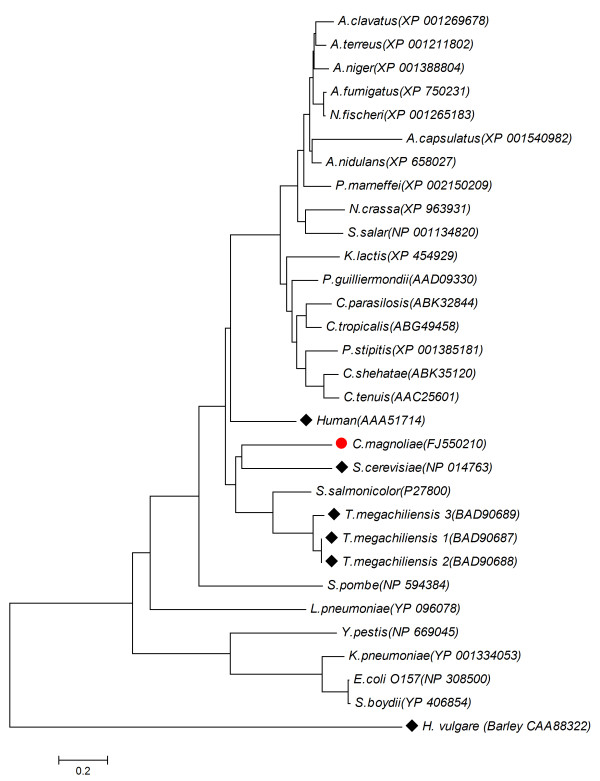
**Phylogenetic analysis of the ER from *C. magnoliae *JH110**. The phylogenetic tree was constructed based on the alignment of the full length amino acid sequences of AKRs including currently known erythrose reductases. AKRs are identified by their GenBank accession numbers. The AKRs used for multiple alignment analysis in Figure 1 are indicated by diamond symbols (black diamond).

By utilizing the recently constructed expressed sequence tag (EST) library of *C. magnoliae *JH110 in our lab (unpublished data), we discovered a clone containing the ER-encoding gene from the randomly sequenced 912 EST clones. To investigate the existence of introns in this EST clone, PCR was performed using the *C. magnoliae *JH110 genomic DNA as a template. The full-sequenced PCR product had exactly the same sequence of the EST clone, indicating absence of intron. A 1041-bp *C. magnoliae *JH110 ER (CmER) with 5'- and 3'-untranslated regions was obtained from the *C. magnoliae *JH110 genomic DNA and it harbored an open reading frame (ORF) of 849 bp with an ATG initiation codon and a TGA termination codon. This gene encodes a polypeptide of 282 amino acid residues with a predicted molecular mass of 31.4 kDa and an isoelectric point of 6.25. The deduced amino acid sequence of the intronless CmER gene was compared with other protein sequences of aldo-keto reductase (AKR) available from the NCBI database using the BLASTP program. The CmER showed a significant homology to the AKR superfamily. The level of identity showed the highest with aldehyde reductase I from *Aspergillus fumigatus *(XP 754700, 44% identity), and ER1 (BAD90687, 42% identity) and ER2 (BAD90688, 41% identity) from *T. megachiliensis *SNG-42. In addition, it was also highly similar to hypothetical protein sequence (XP 662433, 43% identity), which was annotated as AKR in the genome sequence of *Aspergillus nidulans*. The CmER exhibited no similarity to the well-studied xylose reductases among AKR superfamily. The multiple-sequence alignment analysis conducted using aldose reductases with erythrose reduction activity showed two conserved motifs, GYRH and AYSPL, from yeast to human (Fig. 1). The deduced amino acid sequence of the putative CmER gene was used for the construction of a phylogenetic tree with full length amino acid sequences of AKRs from various organisms [19]. In the phylogenetic tree, the CmER was close to the well-characterized glycerol dehydrogenase coded by *GCY1 *from *Saccharomyces cerevisiae*, aldehyde reductase from *Sporobolomyces salmonicolor*, and ERs from *T. megachiliensis *SNG-42. Furthermore, the CmER formed a family in the AKR clades separated from other known AKR clades. This result indicates that CmER has evolved differently from the other AKR family members (Fig. 2).

### Cloning, heterologous expression, and purification

**Figure 3:**
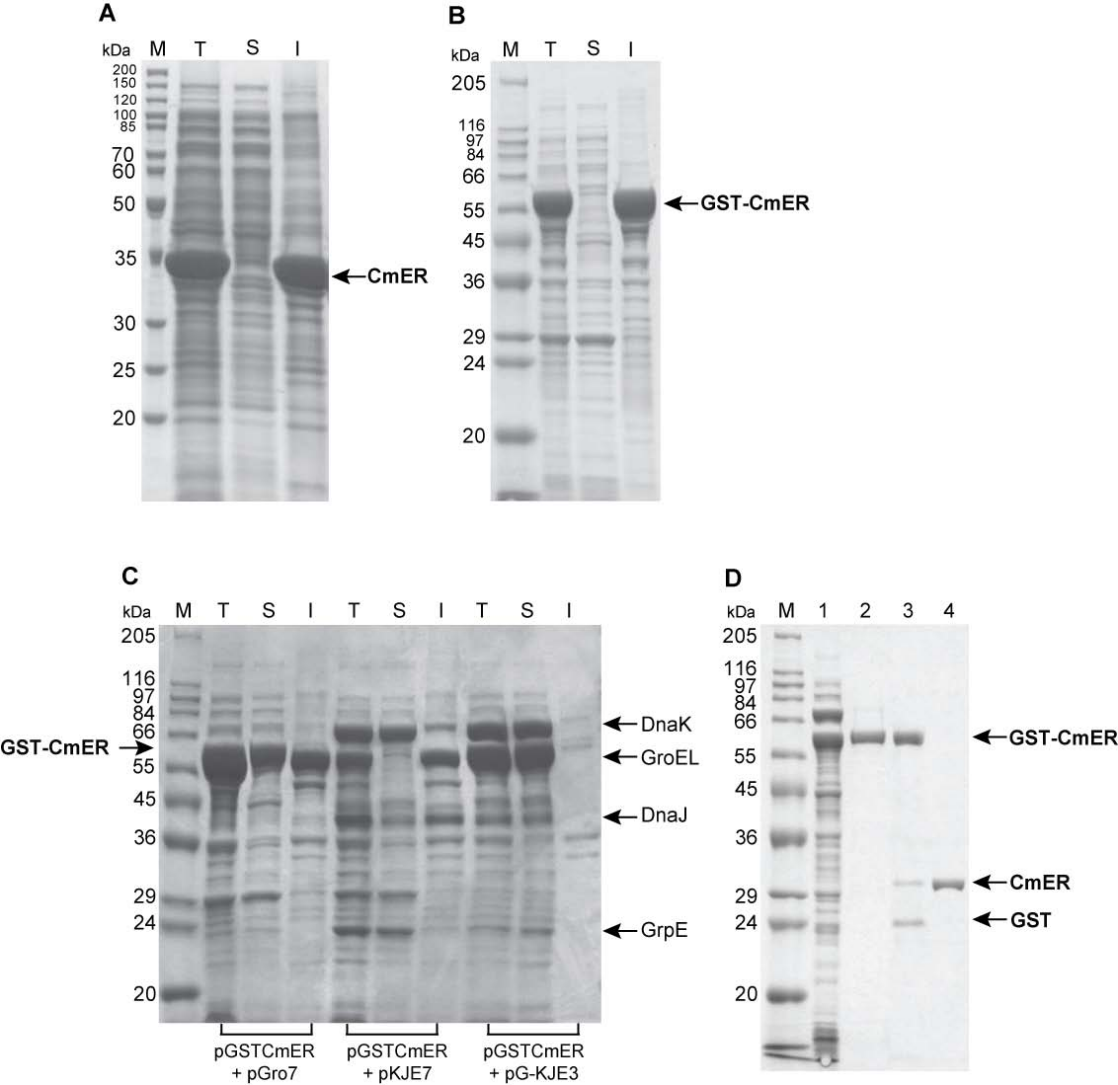
**SDS-PAGE analysis of CmER expressed in recombinant *E. coli***. (A) Expression of CmER in the transformant *E. coli *BL21(DE3) harboring pCmER10, (B) Expressed CmER fused to GST at its N-terminus under the control of the *tac *promoter in *E. coli *BL21, (C) CmER expressed from the GST-fusion approach in conjunction with co-expression of molecular chaperones; Plasmids encoding the molecular chaperone, pGro7, pKJE7, and pGKJE3, were used to express GroEL-GroES, DnaK-DnaJ-GrpE, and GroEL-GroES-DnaK-DnaJ-GrpE, respectively, (D) SDS-PAGE analysis of CmER expressed and purified from the GST-fusion approach in conjunction with co-expression of molecular chaperones, GroEL-GroES-DnaK-DnaJ-GrpE. Lane M, protein standards; lane T, IPTG-induced total fraction; lane S, soluble fraction; lane I, insoluble fraction. Lane 1, soluble fraction of GST-fused CmER-expressed cells; lane 2, purified GST-CmER fusion protein; lane 3, cleavage of GST from GST-CmER fusion protein by Factor Xa; lane 4, purified intact CmER.

To express CmER in recombinant *Escherichia coli*, the transformant *E. coli *BL21(DE3) harboring pCmER10 was induced with 1 mM isopropyl β-D-1-thiogalactopyranoside (IPTG) at 37°C. After IPTG induction for 6 h, a protein of an apparent 33 kDa molecular weight appeared as a major band in sodium dodecyl sulfate polyacrylamide gel electrophoresis (SDS-PAGE) analysis (Fig. 3A). Even though an overall expression level was quite high, the protein was produced mainly as inclusion bodies. In order to express the soluble and active form of CmER in *E. coli*, the fusion gene approach was attempted. Glutathione S-transferase (GST) is a naturally-occurring 26 kDa protein which is often used for the soluble expression of heterologous proteins [20]. In addition, the GST fusion tag with integrated protease sites has been used for convenient purification of the proteins of interest. Thus, the CmER fused to GST at its N-terminus was expressed under the control of the *tac *promoter by 1 mM IPTG induction in *E. coli *BL21, which also resulted in the formation of inclusion bodies (Fig. 3B). Since it has been reported that the co-expression of molecular chaperones increases the soluble form of several enzymes in *E. coli *[21,22], their effects on CmER expression were tested in the transformant *E. coli *cells harboring pGSTCmER. Plasmid pGro7, pKJE7, or pG-KJE3 [23] was used for co-expression of GroEL-GroES, DnaK-DnaJ-GrpE, or GroEL-GroES-DnaK-DnaJ-GrpE, respectively. Plasmid pGro7, pKJE7, or pG-KJE3 was simultaneously transformed with the plasmid pGSTCmER into *E. coli *BL21 cells. Among the chaperone families co-expressed, only the GroEL-GroES-DnaK-DnaJ-GrpE chaperone family exerted positive effects on soluble expression of CmER as verified by SDS-PAGE (Fig. 3C). After confirming soluble enzyme expression, the GST-removed CmER was purified by using the affinity column chromatography after cleavage with Factor Xa. The enzyme purity was estimated by SDS-PAGE analysis with staining of Coomassie brilliant blue. The final yield of intact CmER was 18 mg (~60 mg/liter of culture) of >95% pure CmER with a molecular mass of ~31 kDa, which corresponds well to the calculated mass (Fig. 3D).

### Steady-state kinetics

Most of AKRs described to date are pyridine nucleotide linked, requiring either NADH or NADPH as a coenzyme. The purified CmER had activity only with NADPH (Table 1). The K_m _value for NADPH was determined to be 0.016 ± 0.01 mM. In order to study the substrate specificity of the purified CmER, a series of aldoses with different numbers of carbon atoms (C4 - C6) and aromatic aldehyde *p*-nitrobenzaldehyde were tested with NADPH in excess. Kinetic parameters of the sugar substrates tested for CmER is summarized in Table 1. As expected, the CmER displayed the highest activity toward D-erythrose in the presence of NADPH. This substrate was reduced to the corresponding alcohol, erythritol. Very low activities were found with D-xylose and D-ribose at concentration as high as 200 mM and no activity was detected with D-glucose, D-galactose, and D-fructose, which is consistent with other characterized ERs [14,24]. Moreover, the CmER shows the greatest preference for D-erythrose based on the specificity constant (k_cat_/K_m_), and the k_cat _and k_cat_/K_m _values are considerably higher for NADPH than for NADP^+ ^(Table 1).

**Table 1:** Steady-state kinetics for *C. magnoliae *JH110 ER

Substrate	Specific activity (U/mg protein)	**K**_**m **_**(mM)**	**k**_**cat **_**(S**^**-1**^**)**	**k**_**cat**_**/K**_**m **_**(mM**^**-1 **^**S**^**-1**^**)**
*p*-Nitrobenzaldehyde	4.3 ± 0.3	0.17 ± 0.01	1.4 ± 0.1	8.2
D-Erythrose	16.5 ± 1.3	8.5 ± 0.4	7.6 ± 0.4	0.89
D-Xylose	0.96 ± 0.06	72 ± 3.4	1.8 ± 0.1	0.011
D-Ribose	0.66 ± 0.01	56 ± 4.1	1.7 ± 0.2	0.030
D-Glucose	N.D.	N.D.	N.D.	N.D.
D-Fructose	N.D.	N.D.	N.D.	N.D.
D-Galactose	N.D.	N.D.	N.D.	N.D.
NADH	N.D.	N.D.	N.D.	N.D.
NADP^+^	6.4 ± 0.5	0.24 ± 0.04	0.84 ± 0.06	3.5
NADPH	81.3 ± 2.7	0.016 ± 0.01	48 ± 2.5	3000

N.D.; Not Detected.

### Temperature and pH dependence

**Figure 4:**
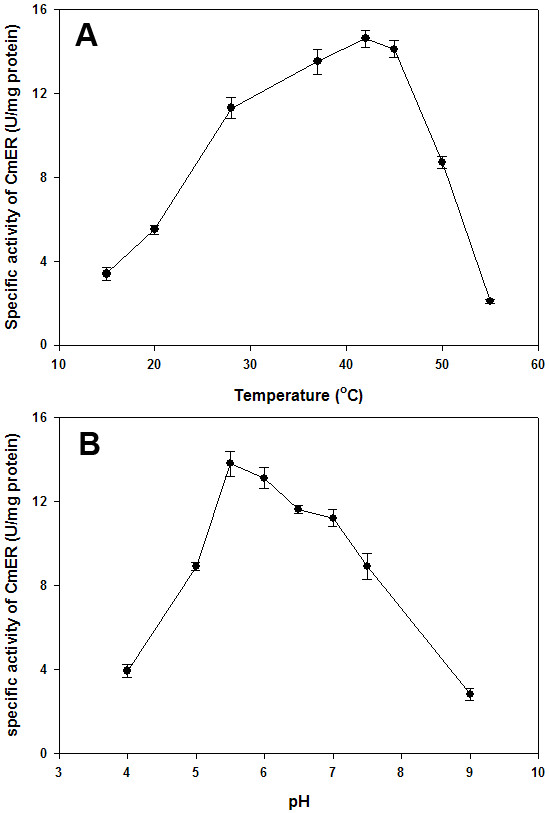
**Determination of pH and temperature optima**. (A) Effect of temperature on the specific activity of the CmER. For the effect of temperature, the activity of CmER was determined at different temperatures between 15 and 55°C at pH 5.5. (B) pH dependence of CmER activity. The effect of pH on the activity of CmER was determined by assaying the enzyme activity at various pH values (pH 4.0 - 9.0). The reactions were carried out at 37°C in the following buffer systems and pH values: sodium citrate buffer (pH 4.5 - 6.0), potassium phosphate buffer (pH 6.0 - 8.0), Tris-HCl (pH 8.0 - 9.0), and glycine-NaOH buffer (pH 9.0 - 10.0). The assays were performed in triplicate at each temperature and pH point.

The activity of the CmER was maximum at 42°C when assayed under standard conditions at temperatures ranging from 15 to 55°C (Fig. 4A). The activity of the CmER as a function of the pH value and the buffer system was studied between pH 4.0 and 9.0. The optimal pH was observed at 5.5 in sodium citrate buffer (Fig. 4B) and only about 20% of maximal activity was observed at pH of ≤ 4.0 and ≥ 9.0.

### Homology modeling

**Figure 5:**
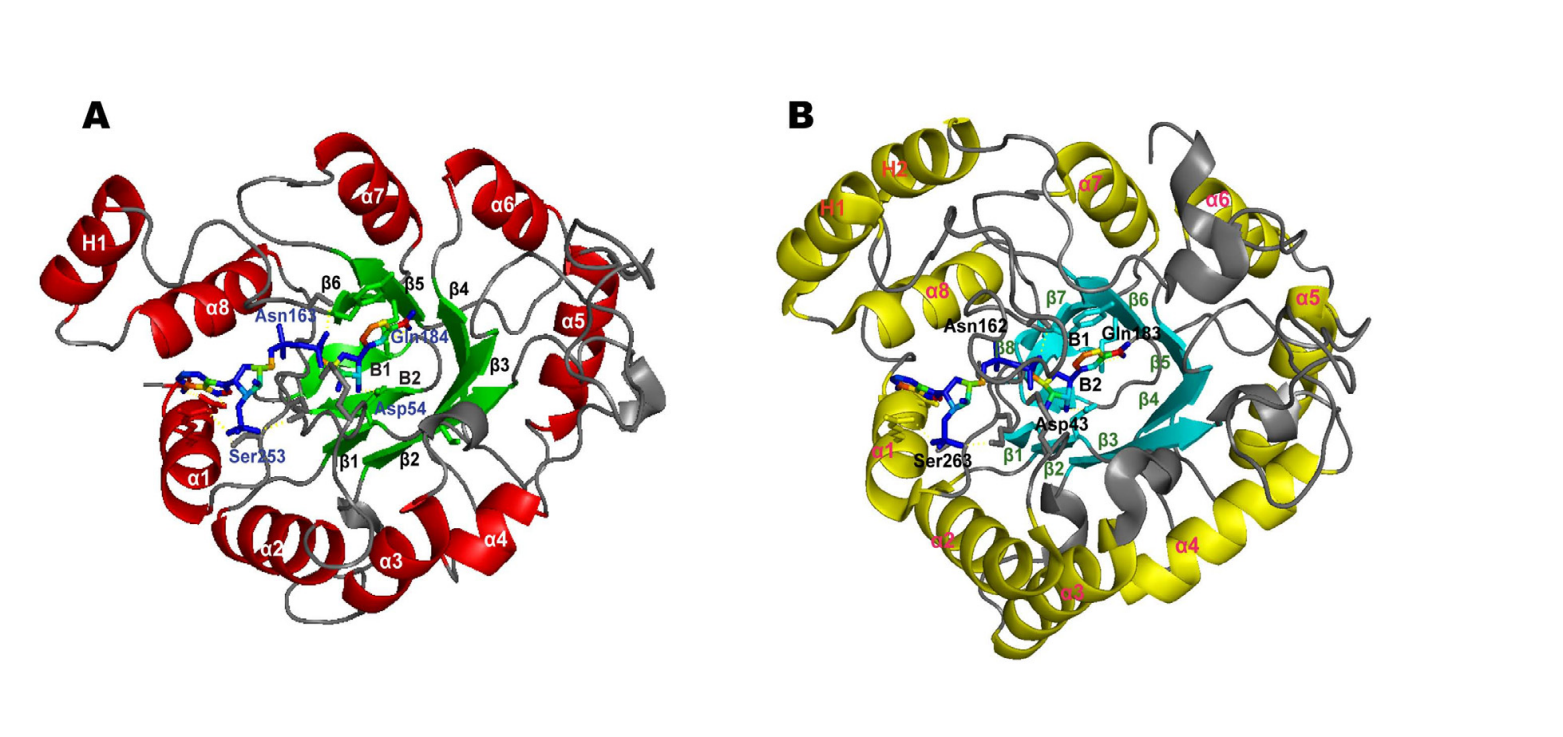
**Comparison of the crystal structure of homology model for *C. magnoliae *JH110 ER (A) with barley ALR1 (B)**. Looking down at the NADPH cofactor in the active site at the center of the (α/ß)_8_-barrel. A hairpin (B1 + B2) covers one end of the barrel while a NADPH binding site is sealing the opposite end. Helices are red (CmER) and yellow (barley ALR1), and sheets are green (CmER) and cyan (barley ALR1). The cofactor is included in sticks. Amino acids bound to the NADPH were colored by blue (CmER) and black (barley ALR1).

Using the coordinates (Protein Data Bank http://www.pdb.org) for aldose reductase 1 (ALR1) from barley (PDB accession code, 2BGS) and human aldose reductase (PDB accession code, 1HQT), which have the erythrose reduction activity; a homology model docked with NADPH was created. Unlike other AKR enzymes, the resulting CmER model is an (α_8_/β_6_) barrel oxidoreductase with only one additional α-helix (H1). A hairpin (B1 + B2) covers one end of the barrel while a NAPDH binding site is sealing the opposite end. The H1-helix represents a junction between strand β6 and helix α7. However, the overall fold and binding of coenzyme were similar to the barley ALR1 crystal structure, as depicted in Fig. 5. The conserved catalytic residues, Trp31, Tyr60, His121, and Trp122 (CmER residue number), from barley ALR1 have similar orientations and locations in the CmER model. Structures of other AKR superfamily members display similar interactions with NADPH. In CmER, Asp54, Asn163, Gln184, and Ser253 (CmER residue number) are the NADPH hydrogen bond partners that are completely conserved in the superfamily [25]. Furthermore, NADPH interacts with the side chains of Lys252, Thr255, and Arg258 in the CmER, which could account for the enzyme's absolute requirement of NADPH over NADH [24].

### Discussion

We have isolated and characterized the CmER gene that encodes erythrose reductase from *C. magnoliae *JH110. AKRs, such as aldehyde reductase and carbonyl reductase, from *C. magnoliae *strain have been reported [26,27] and there are reports on the purification and properties of ERs from only three other yeasts [14,15,28]. However, this is the first report on the entire primary structure of the ER gene from *C. magnoliae *JH110, a microorganism with the potential for industrial erythritol production.

Initial expression of the CmER in *E. coli *resulted in formation of inclusion body. However, the efficiency of soluble CmER expression was improved using the combination of GST gene fusion and molecular chaperone co-expression (Fig. 3C). Molecular chaperones are known to play a role in protecting proteins from aggregation of unfolded or partially folded proteins in cells. It was also reported previously that co-expression of the DnaK-DnaJ-GrpE and GroEL-GroES molecular chaperone complexes improved proper folding of product proteins in *E. coli *[23]. In addition, expression of GroEL-GroES may cooperate with DnaK-DnaJ-GrpE in a synergistic way to increase soluble production of some proteins, indicating that they play cooperative roles in protein folding [23]. In agreement with this synergistic effect, soluble expression of CmER was resulted from the co-expression of GroEL-GroES-DnaK-DnaJ-GrpE, i.e., co-expression of single co-expression system (GroEL-GroES or DnaK-DnaJ-GrpE) was not as effective as its combined co-expression system.

Although some AKRs utilize both cofactors, NADH and NADPH [29,30], the CmER showed no activity in the presence of NADH (Table 1). This is consistent with the fact that the AKRs generally have a greater affinity for NADPH than NADH in mammalian tissues and yeasts [30,31]. Kinetic characteristics of purified and characterized ERs from *C. magnoliae *KFCC-11023, *S. cerevisiae*, *Torula corallina, T. megachiliensis *SNG-42, *Schizophyllum commune*, and barley are compared in Table 2. The CmER has a higher k_cat _and higher catalytic efficiencies (k_cat_/K_m_) with respect to erythrose and NADPH. Its catalytic efficiency with respect to NADPH was 333-fold higher than that of the next closest enzyme [24]. The K_m _value for D-erythrose (8.5 mM) is comparable to the values for the ERs from *T. megachiliensis *SNG-42 [15], *T. corallina *[28], and *S. commune *[32], which have K_m_s of 8.2, 7.1, and 5.0 mM, respectively. In addition, the NADPH-dependent erythrose reduction was detected whereas NADP^+^-dependent erythritol oxidation were not observed, suggesting that under physiological conditions the formation of erythritol is thermodynamically favorable in *C. magnoliae *JH110 and hence the CmER has been better adapted to erythritol biosynthesis but not to the utilization of erythritol.

**Table 2:** Properties of ER from various organisms

ER* (reference)	Molecular mass (kDa)		Optimum		Erythrose			NADPH		
	
	Subunit	Native	pH	Temp. (°C)	**K**_**m **_**(mM)**	**k**_**cat **_**(S**^**-1**^**)**	**k**_**cat**_**/K**_**m **_**(mM**^**-1 **^**S**^**-1**^**)**	**k**_**cat **_**(S**^**-1**^**)**	**K**_**m **_**(mM)**	**k**_**cat**_**/K**_**m **_**(mM**^**-1 **^**S**^**1**^**)**
CmER2	31	N.R.	5.5	42	8.5	7.6	0.89	48	0.016	3000
CmER1 [18]	38.8	79.0	7.0	N.R.	7.9	5.7	0.73	0.013	450	0.66
BaALR1 [24]	34	34	N.R.	N.R.	45	2.3	51	1.5	0.2	9.0
TcER [17]	35.4	71.0	6.0	40	7.12	N.R.	N.R.	N.R.	N.R.	N.R.
ScALR [32]	N.R.	N.R.	7.0	N.R.	5.0	N.R.	N.R.	N.R.	N.R.	N.R.
ScGCY1 [49]	35	N.R.	N.R.	N.R.	3.4	N.R.	N.R.	N.R.	N.R.	N.R.
TmER1 [15]	38	38	6.5	45	7.1	N.R.	N.R.	N.R.	N.R.	N.R.
TmER2 [15]	37	37	6.5	45	7.6	N.R.	N.R.	N.R.	N.R.	N.R.
TmER3 [15]	37	37	6.5	45	8.2	N.R.	N.R.	N.R.	N.R.	N.R.

*CmER2, *C. magnoliae *JH110 (This study); CmER1, *C. magnoliae *KFCC-11023; BaALR1, Barley; TcER, *T. corallina*; ScALR, *Schizophyllum commune*; ScGCY1, *S. cerevisiae*; TmER1,2, and 3, *T. megachiliensis *SNG-42. N.R.; Not Reported.

The CmER gene heterologously expressed and characterized here does not appear to encode an ER previously isolated and characterized from *C. magnoliae *KFCC-11023, which is a chemical mutant of *C. magnoliae *JH110. Due to the lack of genetic and sequence information of ER from *C. magnoliae *KFCC-11023, it is not certain to what degree they are different, but there are several key differences between the two proteins. The molecular weights and apparent kinetic constants are different (Table 1 and 2). Additionally, the previously isolated ER showed dual cofactor activity with NADH and NADPH and preferred NADH as the cofactor. The two enzymes differ in their pH optima and K_m _values for D-erythrose. The enzyme, which possessed ER activity from *C. magnoliae *KFCC-11023, has been reported to have the KXXXGF(Y/G)XG motif [18]. This motif is conserved in the xylose reductase subfamily, but is not found among currently known ERs in *T. megachiliensis *SNG-42 and *C. magnoliae *JH110. Although these differences could be due to alternative mRNA splicing, posttranslational modifications in *C. magnoliae *JH110, or the fusion of the GST tag, the multiplicity of ERs has been reported in the yeast [15]. Thus, the most logical conclusion is that these are different proteins from different genes.

Barley ALR1 exhibits 15- to 30-fold higher specific activity toward short monosaccharides, for example DL-glyceraldehyde (C3) and D-erythrose (C4), than toward the osmoregulatory-relavant substrates such as xylose (C5) and glucose (C6) [24]. The barley ALR1 binds with NADPH and does not react with NADH [24]. Structural models of barley ALR1 suggested that Tyr49 acts as the acid base catalyst, and Asp44, Lys79, and His112 play an important role in facilitating the hydride transfer [33,34]. All four of these amino acid residues are conserved throughout the superfamily, including the CmER. These enzymes have a strict requirement for NADPH. The tetra-amino acid motif, IPKS, is conserved among these NADPH-dependent reductases and the lysine residue in this motif is involved in NADPH binding [35]. Although the motif (LPKS) is present in the CmER, some amino acid residues were not conversed around the motif. The crystal structures of aldose reductases bound with NADPH or an analog of the cofactor show that the enzyme undergoes a large conformational change upon binding with NADPH. The change involves the reorientation of loop 7 to a position which appears to lock the coenzyme into place [36,37]. The putative loop 7 in ER from *C. magnoliae *JH110 is shorter than those in other AKRs. The K_m _values for NADPH were estimated to be within the range of 2 to 4 μM for mammalian reductases [31,38] and 37.5 μM for *S. salmonicolor *reductase [39]. The difference in the K_m _values could be due to an amino acid sequence difference around the NADPH-bind region. Most AKRs have a profound preference for NADPH, but remain functional with NADH [40,41]. In *Candida tenuis *XR (CtXR), which has a modest 33-fold preference for NADPH over NADH, a structural rearrangement occurs in the active site in response to the preference of either NADPH or NADH [42]. A similar rearrangement is considered impossible in CmER, not only because of its short B-loop but also due the small side chains in the B and β_6_-α_8 _loops (Gly216 and Ser254 in CmER versus Glu227 and Asn267 in CtXR).

### Conclusions

This study provides a novel ER from *C. magnoliae *JH110, which is one of the most efficient erythritol producers. The *C. magnoliae *JH110 ER specifically acts on the erythrose with highly conserved NADPH binding site. The ease of isolating this enzyme coupled with its high activity and catalytic efficiency may be useful in the *in vitro *production of erythritol. The application value of this enzyme will be tested with *in situ *cofactor regeneration and heterologous expression in other microorganisms, which do not produce erythritol.

### Methods

#### Chemicals and enzymes

*P*-Nitrobenzaldehyde, D-erythrose, D-ribose, D-xylose, D-fructose, D-galactose, D-glucose, and enzyme cofactors (NADH and NADPH) were purchased from Sigma-Aldrich (St. Louis, MO). Restriction endonucleases and other cloning reagents were purchased from New England Biolabs (Beverly, MA). The DNA samples were purified using the AccuPrep PCR purification kit (Bioneer, DaeJeon, Korea). Oligonucleotides were synthesized by Bioneer. The RediPack column packed with Glutathione Sepharose 4 Fast Flow was obtained from GE Healthcare Bio-Sciences (Uppsala, Sweden). Bacto peptone and yeast extract were from Difco Laboratories (Grayson, GA). The dye reagent for protein assay was from Bio-Rad (Hercules, CA). Unless stated otherwise, all other reagents and chemicals were from standard suppliers.

#### Strains and plasmids

A *C. magnoliae *JH110 wild type strain [Korean Culture Center of Microorganisms (KCCM) (formerly Korean Federation of Culture Collection) (KFCC))-10900] was maintained on YPS agar plate (10 g/L yeast extract, 20 g/L bacto peptone, and 300 g/L sucrose) at 4°C. *E. coli *DH5α was used for all DNA manipulations. *E. coli *BL21 and *E. coli *BL21(DE3) were used as expression hosts grown in Luria-Bertani (LB) medium (5 g/L yeast extract, 10 g/L tryptone, and 10 g/L NaCl) supplemented with appropriate antibiotics when required. Plasmid pETR10 containing the 10 arginine tag was derived from pET29-b(+) (Novagen, Madison, WI) [43] and used for expression of the ER gene in *E. coli *BL21(DE3). An expression vector pGEX-5X-3 (GE Healthcare Bio-Sciences) was used for expression of the ER gene as a GST-tagged fusion protein. Plasmid pGEX-5X-3 possesses a Factor Xa recognition site for cleavage of GST from the target protein. Plasmid encoding the molecular chaperone, pGro7, pKJE7, or pGKJE3 in which the L-arabinose-inducible promoter (*ara *BAD) was used to express GroEL-GroES, DnaK-DnaJ-GrpE, or GroEL-GroES-DnaK-DnaJ-GrpE, respectively, were kindly donated by Dr. Hideki Yanagi (HSP Research Institute, Kyoto Research Park, Kyoto, Japan).

#### Isolation of ER gene from *C. magnoliae *JH110

Random sequencing of the EST clones prepared from *C. magnoliae *JH110 yielded 912 successful sequencing reactions. BLAST analysis of all the ESTs revealed that an EST clone was highly similar to previously identified ER of *T. megachiliensis *SNG-42. This EST clone was named as putative erythrose reductase of *C. magnoliae *JH110 and selected for further cloning. To verify the sequence of the putative CmER gene, the gene-specific primers, ER1 (ATGTCTTCGACCTACACCCTTAC) and ER2 (TCACCGTCTTGCTAGCGC), were designed to amplify the sequence covering the ORF of CmER with 5' and 3' flanking regions. The PCR product was gel-purified and cloned into the pGEM-T Easy vector (Promega, Madison, WI) to facilitate DNA sequencing. After transformation into competent cells of *E. coli *DH5α, the recombinants were identified through blue-white color selection in ampicillin-containing LB-agar plates. The positive clones were sequenced in both directions and the resulting sequences were confirmed.

#### Sequence analysis

The searches for nucleotide and protein sequence similarity were conducted with the BLAST algorithm at the National Center for Biotechnology Information http://www.ncbi.nlm.nih.gov/blast. The deduced amino acid sequences were analyzed with the Expert Protein Analysis System http://www.expasy.org/ and protein domain features of CmER were determined by using the Simple Modular Architecture Research Tool [44]. The deduced amino acid sequence of CmER was aligned with the corresponding AKR sequences including ER from various organisms using the CLUSTALW software [45]. A phylogenetic tree was constructed with MEGA 3.1 software by using the neighbor-joining method [46]. Boostrap analysis was used with 1000 replicates to test the relative support for the branches produced by the neighbor-joining analysis [47]. All the analyzed sequences of aldose reductase enzymes were retrieved from GenBank and SWISS-PROT database.

#### Expression and purification

After identifying the EST sequence of CmER, the CmER gene was amplified from the *C. magnoliae *JH110 genomic DNA by PCR using the primer ER3 (GGAATTC**CATATG**TCTTCGACCTACACC, NdeI restriction site is highlighted as bold) and ER4 (CGC**GGATCC**CCGTCTTGCTAGCGCG, BamHI restriction site is highlighted as bold). The amplified CmER gene was sequenced and compared with the sequence of the identified putative CmER EST clone to investigate the existence of introns. For expression of the CmER gene, the amplified CmER was cloned into plasmid pETR10, resulting in pCmER10. For fusion gene approach, the amplified CmER with a set of primers (ER5; CGC**GGATCC**CCTCTTCGAACCTACACCCTTACTCG, BamHI restriction site is highlighted as bold and ER6; ATAAGAAT**GCGGCCGC**TCACCGTCTTGCTAGCGC, NotI restriction site is highlighted as bold) was inserted into plasmid pGEX-5X-3 to express the GST-fused CmER, resulting in pGSTCmER. The constructed recombinant vectors, pCmER10 and pGSTCmER, were introduced into *E. coli *BL21 (DE3) and *E. coli *BL21 cells, respectively. Transformant cells were selected on LB-agar plates containing appropriate antibiotics and cultured in LB media at 37°C with vigorous shaking. Expression of the CmER and molecular chaperone genes were induced at the logarithmic phase of growth (OD_600 _≈ 0.5) by adding IPTG and L-arabinose to a final concentration of 1 mM and 1 g/L, respectively. The cells induced for 6 h were then harvested by centrifugation at 6,000 × g for 20 min at 4°C and resuspended in 1 × PBS buffer (pH 7.4) containing the protease inhibitor cocktail (Sigma-Aldrich) for disruption by sonication. The crude extract was fractionated into soluble and insoluble fractions by centrifugation at 20,000 × g for 30 min at 4°C. These fractions were analyzed by SDS-PAGE and the soluble fraction was used for the subsequent purification of the CmER enzyme. ÄKTA FPLC (GE Healthcare Bio-Sciences), an automated chromatography system, was used to analyze the separation pattern of the fusion protein. The soluble fraction was directly applied to the GSTrap FF column (1 mL, GE Healthcare Bio-Sciences) prepacked with Glutathione Sepharose 4 Fast Flow (GE Healthcare Bio-Sciences). The column was washed with 1 × PBS buffer (pH 7.4) at a flow rate of 0.5 mL/min. After washing, on-column cleavage was performed using Factor Xa dissolved in cleavage buffer (1 mM CaCl_2 _and 150 mM NaCl in 50 mM Tris-HCl, pH 7.5) and then the bound protein was eluted. For removal of Factor Xa protease, the eluate was passed directly into the column manually packed with Benzamidine 4 Fast Flow (GE Healthcare Bio-Sciences). The final eluate without Factor Xa was analyzed for the SDS-PAGE and enzymatic assay.

#### Determination of protein content and enzyme activity

Protein content was determined using a protein assay kit (Bio-Rad) with bovine serum albumin as standard. The CmER activity was determined as reported previously [14] with modifications given below. Five microliter of the purified enzyme solution was incubated in 250 μL of 50 mM phosphate buffer (pH 6.5) containing 12 mM D-erythrose and 4 mM NADH or NADPH at 37°C for 10 min. Immediately after the reaction, the absorbance at 340 nm was measured by an Ultrospec 4000 spectrophotometer (GE Healthcare Bio-Sciences). One unit of enzyme activity was defined as the amount of enzyme which produced 1 μmol of NAD(P)^+ ^per minute under the above conditions.

#### Effects of pH and temperature

The influence of pH on the activity of CmER was studied by assaying the enzyme activity at various pH values (pH 4.0 - 9.0). The reactions were carried out at 37°C in the following buffer systems and pH values: sodium citrate buffer (pH 4.5 - 6.0), potassium phosphate buffer (pH 6.0 - 8.0), Tris-HCl (pH 8.0 - 9.0), and glycine-NaOH buffer (pH 9.0 - 10.0). D-Erythrose was a variable substrate and NADPH remained at a fixed concentration of 50 μM. For the effect of temperature, the activity of CmER was determined at different temperatures between 15 and 55°C at pH 5.5. The assays were performed in triplicate at each temperature and pH point.

#### Steady-state kinetics

Kinetic parameters of purified CmER were determined by steady-state kinetics experiments. The enzyme reaction for D-erythrose reduction was performed at optimal pH 5.5 and temperature 42°C by varying concentrations of one substrate (D-erythrose or NADPH) while the other was kept constant under saturated conditions. The kinetic parameters, K_m _and V_max_, were determined by plotting the reaction rates against substrate concentrations to fit the Michaelis-Menten kinetics.

#### SDS-PAGE analysis

SDS-PAGE was performed according to Laemmli [48] using a Mini PROTEAN 3 electrophoresis system (Bio-Rad). The gels were visualized by staining with Coomassie brilliant blue R-250.

#### Homology modeling

Molecular Operating Environment (MOE, Chemical Computing Group Inc., Montreal, Canada) was used to prepare and optimize the model and Insight II (version 2000; Accelrys Inc., San Diego, CA) was used for analysis. To verify the model, the overall fold was checked using Profiles-3D (Insight II), and the allowed states for φ and ψ angles and bond distances were checked using ProStat (Insight II), both with default settings. The Profiles-3D check resulted in a self-compatibility score of 86.5, which compares well to the scores of 98.3, 93.5 and 106.2 for the coordinates from 1ADS, 1BGS and 1HQT, respectively. The ProStat check of φ and ψ angles were determined to be 75.2% within the allowed Ramanchandran region, comparing well to the 87.1%, 89.4 and 84.6% for the same analysis of PDB structures 1ADS, 1BGS, and 1HQT, respectively and lying in the core region of the Ramachandran plot. Therefore, our calculated results imply that the modeled structure of CmER is reliable.

#### Nucleotide sequence accession number

The nucleotide sequence of genomic CmER gene has been submitted to the GenBank database under accession number FJ550210.

### Competing interests

The authors declare that they have no competing interests.

### Authors' contributions

JHS organized funding, helped to draft the manuscript, and coordinated the study. YWR coordinated the project. DHL drafted the manuscript and participated in the design and coordination of the study. YJL drafted the manuscript. All authors have read and approved the final version of the manuscript.
